# Cutaneous Adverse Events Associated with Semaglutide, Liraglutide and Tirzepatide: A Systematic Review

**DOI:** 10.3390/medsci14060652

**Published:** 2026-10-09

**Authors:** Aditya K. Gupta, Elizabeth M. Teasell, Renata F. Magalhães, Paradi Mirmirani, Vincent Piguet

**Affiliations:** 1 Division of Dermatology, Department of Medicine, Temerty Faculty of Medicine, University of Toronto, Toronto, ON M5S 1A8, Canada; vincent.piguet@utoronto.ca; 2 Division of Dermatology, Women’s College Hospital, Toronto, ON M5S 1B2, Canada; 3 Independent Researcher, London, ON N5X 2P1, Canada; 4 Dermatology Division, School of Medical Science, Universidade Estadual de Campinas, Campinas 13083-894, Brazil; renatafm@unicamp.br; 5 Department of Dermatology, The Permanente Medical Group, Vallejo, CA 94589, USA; paradi.mirmirani@kp.org; 6 Department of Dermatology, University of California, San Francisco, CA 94115, USA

**Keywords:** GLP-1 receptor agonist, GIP/GLP-1 receptor agonist, skin and subcutaneous adverse events, dermatology, dysesthesia, injection-site reaction, rash

## Abstract

Background/Objectives: Incretin-based therapies, including glucagon like peptide-1 receptor agonists (GLP-1 RAs) and glucose-dependent insulinotropic polypeptide (GIP)/GLP-1 RAs, are increasingly prescribed for obesity, leading to greater recognition of associated dermatological adverse events (AEs). We aimed to characterize the spectrum, frequency, and clinical characteristics of cutaneous AEs reported with semaglutide, liraglutide and tirzepatide, focusing on these agents because of their established use in both weight management and diabetes mellitus. Methods: A systematic literature search was conducted in MEDLINE (via PubMed), Embase, and Web of Science. Search terms combined skin-related manifestations (e.g., skin, dermatologic, injection-site reactions, pruritus, rash, hypersensitivity, dysesthesia, vasculitis) with GLP-1 RA and GIP/GLP-1 RAs (semaglutide, tirzepatide, liraglutide). Clinical trials, observational studies, pharmacovigilance studies, and case reports reporting skin-related AEs associated with semaglutide, liraglutide and tirzepatide were included. Results: One hundred and one studies were included (32 randomized controlled trials, 6 retrospective cohort/cross-sectional studies, 20 pharmacovigilance studies, and 43 case series/reports). Cutaneous AEs ranged from generally common but mild reactions to rare but potentially severe immune-mediated disorders. Injection-site reactions, including erythema, pruritus, and hemorrhage, were reported most frequently with tirzepatide (2–7%), then liraglutide, and less commonly with semaglutide, with evidence of dose dependence. Dysesthesia was most strongly associated with semaglutide, occurring in approximately 5% of patients at standard doses and up to 20% with high-dose formulations indicated for weight management, with events frequently occurring during dose escalation. Uncommon reactions reported in association with GLP-1 or GIP/GLP-1 RA use included angioedema, hypersensitivity reactions, autoimmune blistering diseases, drug eruptions, vasculitis, and severe cutaneous reactions. Conclusions: Although most cutaneous AEs associated with GLP-1 and GIP/GLP-1 RAs are mild and manageable, clinically relevant drug-specific differences exist. The recognition of common and uncommon skin manifestations may facilitate earlier diagnosis, support treatment adherence, and reduce the adverse outcomes.

## 1. Introduction

Incretin-based therapies, including glucagon-like peptide-1 receptor agonists (GLP-1 RAs) and dual glucose-dependent insulinotropic polypeptide/glucagon-like peptide-1 receptor agonists (GIP/GLP-1 RAs), have transformed the management of type 2 diabetes mellitus (T2DM) and obesity. Originally approved for glycemic control in T2DM, GLP-1 RAs such as semaglutide, liraglutide, and the GIP/GLP-1 RA tirzepatide, have since demonstrated substantial benefits for weight reduction, leading to expanded regulatory approvals and widespread clinical adoption for weight management [1,2,3,4]. In the United States, the use of GLP-1 and GIP/GLP-1 RAs accelerated around 2023 and has continued to increase since then, with tirzepatide and semaglutide making up over 95% of prescriptions [5]. Although anti-diabetic use is the most common prescribing indication, anti-obesity prescribing is quickly increasingly, exhibiting an overall greater increase than anti-diabetic use, especially among first-time users of semaglutide, such that anti-obesity prescribing of semaglutide and tirzepatide is almost on par with anti-diabetic prescribing [5]. In addition to accelerating demand for these agents across several countries, there has been a surge in indiscriminate, non-prescribed use which may increase the potential risks associated with unsupervised use [6,7].

The safety profile of GLP-1 and GIP/GLP-1 RAs is generally favorable, with mild gastrointestinal symptoms—including nausea, constipation, diarrhea, vomiting, and dyspepsia—being the most frequently reported [8]. Adverse events (AEs) relating to the skin are increasingly recognized and appear to differ between individual agents [7,9,10,11,12]. This systematic review aims to characterize the direct drug-related cutaneous AEs associated with GLP-1 and GIP/GLP-1 RAs used for weight management, including the spectrum and frequency of reported AEs and differences among individual agents. Weight-loss-associated cutaneous changes (i.e., facial aging, cutaneous atrophy, and skin laxity) [13] are distinct from direct drug-related cutaneous AEs and were therefore not the primary focus of this review. Alopecia has recently been comprehensively reviewed [14] and is not the subject of this report.

## 2. Materials and Methods

A systematic search was undertaken across MEDLINE via PubMed, Web of Science, and Embase up to 18 June 2026 following PRISMA guidelines. The review was registered in PROSPERO [CRD420261450018] and a protocol can be found with the registration. The following strategy was used: (cutaneous OR skin OR dermatol* OR “injection-site” OR pruritus OR eczema OR bullous OR vasculitis OR eruption OR angioedema OR rash OR hypersensitivit* OR allodynia OR hyperhidrosis OR urticaria OR dysesthesia OR hyperesthesia) AND (“GLP-1 receptor agonist*” OR “glucagon-like peptide-1 receptor agonist*” OR “GLP-1 agonist*” OR “GLP-1 RA*” OR semaglutide OR tirzepatide OR liraglutide). Human study limits were applied in Embase. The review was restricted to articles published in the English language. Articles were title/abstract screened and full-text reviewed using Covidence 2026 by ET; where uncertain in full-text review, another author was consulted (AKG). References of included studies were screened to identify additional potentially eligible studies. Clinical trials were also manually screened for AEs of interest that may not have been captured by the electronic search strategy, including trials in which these AEs were not identified in the title or abstract. Studies were extracted by ET into a tabulated Excel document noting study characteristics (i.e., indication, drug, GLP-1/GIP/GLP-1 RA type and dose, study length, number of participants) and type and count of cutaneous AEs.

### 2.1. Eliglbility Criteria

#### 2.1.1. Population

Studies involving individuals of any age or sex receiving GLP-1 and GIP/GLP-1 RAs approved for obesity and T2DM (semaglutide, tirzepatide, or liraglutide) with primary indication for weight management or T2DM were eligible, whereas studies involving primary indications other than T2DM or weight loss were excluded. Studies with mixed-treatment populations were excluded when data for users of semaglutide, tirzepatide, or liraglutide could not be extracted separately.

#### 2.1.2. Intervention

Studies reporting cutaneous AEs associated with semaglutide, liraglutide, or tirzepatide were eligible, regardless of dose, formulation, or route of administration. Studies evaluating other GLP-1 and GIP/GLP-1 RAs or failing to specify the GLP-1 RAs used or the reported cutaneous AEs were excluded. Studies reporting only nonspecific AE categories (e.g., skin and subcutaneous tissue disorders) without describing a specific cutaneous reaction were also excluded. Allergic reaction reports were not summarized from clinical trials as it was unclear whether these reactions encompassed cutaneous manifestations. Studies focused exclusively on esthetic or cosmetic skin changes, such as skin laxity, rather than direct AEs, were excluded, as these were not the focus; for a recent review see Barone et al., 2026 [13].

#### 2.1.3. Study Design

Randomized controlled trials (RCTs), observational studies, case series, and case reports published in English were eligible, while reviews were excluded. Given the differences in the frequency and quality of evidence across AEs, evidence synthesis was tailored by outcome. Incidence of injection-site reactions was derived primarily from RCTs, with pharmacovigilance studies included to characterize the spectrum of injection-site reactions. Altered skin sensation was similarly synthesized using RCTs and supplemented by pharmacovigilance data. Hypersensitivity and rash/urticaria were also summarized from RCTs. Clinical trial data were used to describe event frequencies and estimate relative risks, whereas pharmacovigilance data were used to identify disproportionality signals and should not be interpreted as incidence or measures of absolute clinical risk. Rare cutaneous AEs were summarized from case series and case reports as these events may not be captured in controlled trials given their rarity; they were summarized narratively.

### 2.2. Risk of Bias

Risk of bias for RCTs was assessed using RoB 2 tool version 22 Aug 2019 [15] (Figure S1). It was determined there was minimal risk of bias in clinical trials. Non-randomized observational studies were assessed using ROBINS-E version March 2024 [16] (Figure S2). Case reports were assessed using the JBI Critical Appraisal Checklist for Case Reports (accessed 21 September 2026) [17] (Table S1). Pharmacovigilance studies based on spontaneous reporting databases were not subjected to formal risk-of-bias assessment because these analyses differ fundamentally from comparative observational studies and are subject to database-specific limitations that are not adequately captured by existing risk-of-bias tools.

### 2.3. Statistical Analyses

Aggregated event counts were used to calculate relative risks across RCTs for injection-site reactions, altered skin sensation and hypersensitivity. Only RCTs with a placebo or insulin control group were included in relative risk calculations, and calculations were performed separately for placebo- and insulin-controlled trials. Aggregate event counts and percentages are reported for all clinical trials evaluating a given dose, including trials without a placebo or insulin control group. When the trials contributing to the overall aggregated incidence differed from those included in the relative risk calculation, the event counts used for the relative risk calculation are reported separately in the table. Relative risks were calculated from 2 × 2 contingency tables in Excel version 1808. Relative risks are presented with a 95% confidence interval; values with a confidence interval excluding 1.0 were considered statistically significant. This approach was intended to summarize the available evidence rather than provide a formal meta-analytic estimate and does not incorporate study-level weighting or model between-study heterogeneity.

## 3. Results

**Figure 1:**
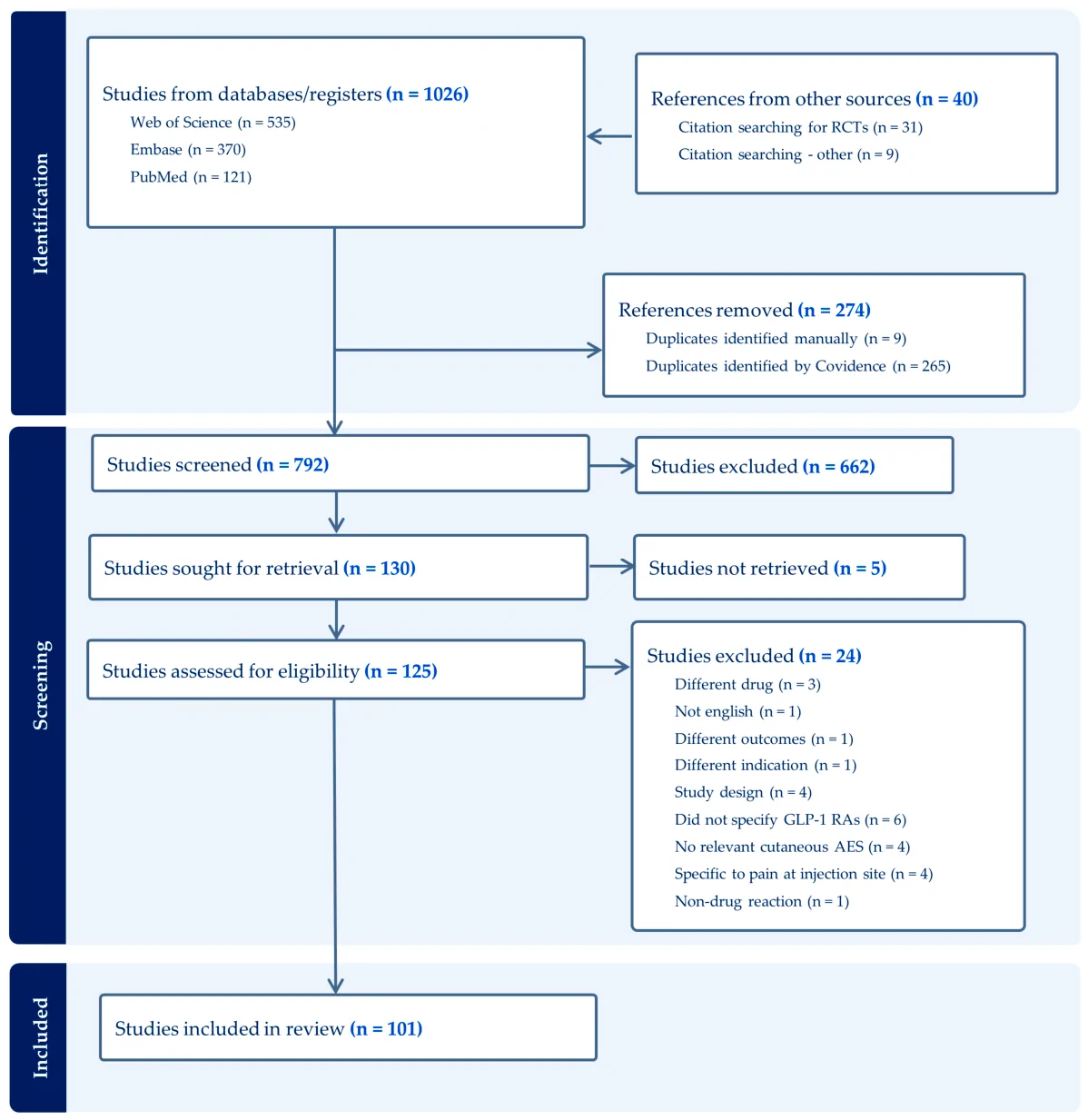
PRISMA chart.

A total of 1026 references were collected across databases and an additional 40 were added from citation searching and screening of relevant RCTs. After screening and full-text review, 101 studies were included, encompassing 32 RCTs, 6 retrospective cohort/cross-sectional, 20 pharmacovigilance studies, and 43 case series/cases reports (Figure 1).

### 3.1. Injection-Site Reactions (ISRs)

Injection-site reactions (ISRs) were among the most frequently reported cutaneous AEs across RCTs, although their frequency varied according to the GLP-1 and GIP/GLP-1 RAs, dose, and treatment indication (Table 1). ISRs include localized AEs occurring at or around the site of subcutaneous injection such as erythema, bruising, hemorrhage, pain, and pruritus.

For tirzepatide, a meta-analysis of clinical trials demonstrated a dose-dependent increase in ISRs, occurring in 1.9%, 2.4%, and 3.1% of patients receiving 5 mg, 10 mg and 15 mg, respectively, compared with the 1.15% receiving placebo, although the individual rates were not statistically different from placebo [9,18]. Relative risks calculated from aggregated event rates were statistically significant across obesity clinical trials for tirzepatide at all doses (5–15 mg) [19,20]; however, in T2DM trials, a statistically significant relative risk was observed only at the 15 mg dose compared to placebo [21,22], while no significant difference was observed compared with insulin [23,24] (Table 1). In clinical trials for obesity and T2DM, ISRs were more frequent among patients who developed anti-tirzepatide antibodies, occurring in 119/2570 (4.63%) of antibody-positive patients compared with 18/2455 (0.73%) of patients negative for antidrug antibodies in T2DM trials [25] and 11.3% of antibody-positive individuals versus 1% of antibody negative individuals in obesity trials [4].

Conversely, in clinical trials, semaglutide was consistently associated with low ISR rates (approximately 1–3%) [26,27], comparable to placebo even at the highest dose evaluated. Aggregate relative risk estimates from included studies demonstrated no significant positive association (Table 1) [26,27]. Evidence further suggests that ISR incidence is higher with tirzepatide compared with semaglutide. In a head-to-head clinical trial in individuals with obesity, ISRs were reported in 8.6% of participants randomized to tirzepatide compared with 0.3% of those receiving semaglutide [10]. Additionally, although ISR rates varied across liraglutide trials, relative risk calculations indicated no positive association with liraglutide compared with placebo at any dose [28,29,30,31,32,33,34,35] (Table 1).

**Table 1:** Injection-site reactions associated with GLP-1 RA-based therapies in RCTs.

GLP-1 & GIP/GLP-1 RA	Incidence (Patients with Adverse Event/Total Taking Medication)						Placebo	Insulin
**Semaglutide**	**1 mg [9]**		**2.4 mg [10,26,30,36,37,38,39,40,41]**		**7.2 mg [26,27]**		**Placebo**	**Insulin**
Obesity w/ or w/o T2DM	1/469 [0.2%]		122/3759 [3.2%] RR_PL_:0.8 [0.64,1.11]		33/1311 [2.5%] RR_PL_:1.5 [0.6,3.88]		87/1894 [4.6%]	—
**Tirzepatide**	**5 mg**	**10 mg**		**10/15 mg**		**15 mg**	**Placebo**	**Insulin**
Obesity [10,19,20]	18/630 [2.9%] **RR_PL_:9.2 [2.1, 39.4]**	36/636 [5.6%] **RR_PL_:18.2 [4.4, 75.3]**		64/661 [9.9%] **RR_PL_:16.3 [3.9, 67.3]**		29/630 [4.6%] **RR_PL_:14.8 [3.5, 61.8]**	4/935 [0.4%]	—
T2DM [9,21,22,23,24,42,43]	26/1701 [1.5%] 8/237 [3.37%] RR_PL_: 7.8 [0.99, 62.4] 2/687 [0.3%] RR_IN_: 0.4 [0.09, 1.8]	37/1702 [2.2%] 7/230 [3.0%] RR_PL_: 6.8 [0.8, 54.8] 8/688 [1.2%] RR_IN_: 1.6 [0.6, 4.0]				55/1714 [3.2%] 11/240 [4.6%] **RR_PL_: 10.7 [1.4,82.1]** 9/686 [1.3%] RR_IN_: 1.8 [0.7, 4.3]	0/113 [0%]	10/1359 [0.7%]
**Liraglutide**	**1.8 mg**			**3 mg**			**Placebo**	**Insulin**
Obesity [28,29,30,31,32,33,34,35]	n/a			226/3046 [7.4%] ^a^ 212/2919 [7.2%] RR_PL_:0.87 [0.7, 1.1]			142/1721 [8.2%] ^a^	—
Obesity + T2DM [44]	34/422 [9.2%] * RR_PL_:0.95 [0.5,1.6]			17/210 [8.1%] * RR_PL_:0.95 [0.5, 1.7]			18/212 [8.5%]	

* Includes reports of injection-site reactions and injection-site hematoma; ^a^ includes two studies in pediatrics of adolescents. Relative risk calculations are presented as relative risk (95% confidence interval). Relative risks where the confidence interval excludes 1.0 are considered statistically significant and bolded. Total incidence numbers were reported across all clinical trials (value underlined); however, only clinical trials with placebo or insulin control groups were included in the relative risk calculations with the incidence values pertaining to these calculations directly above the reported relative risk. GIP, glucose-dependent insulinotropic polypeptide; GLP-1 RA, glucagon-like peptide-1 receptor agonist; T2DM, type 2 diabetes mellitus. RR_PL_, relative risk ratio compared to placebo; RR_IN_, relative risk ratio compared to insulin-degludec or insulin glargine.

Pharmacovigilance studies have provided additional insight into ISRs in real-world settings (Table 2) [7,45,46,47,48,49,50,51,52,53,54]. Females reported ISRs more frequently than males, potentially reflecting both greater use and sex-specific differences in injection technique [47,53]. The median time to onset for ISRs was 8 days [51]. Comparative disproportionality analysis of the Food and Drug Administration Adverse Event Reporting System (FAERS) database through to Q4 2025 found greater reporting odds ratios (RORs) for ISRs with tirzepatide than with semaglutide. Similarly, a disproportionality analysis of VigiBase through 2024 similarly found higher ISR disproportionality signals among tirzepatide users than semaglutide and liraglutide [7,52,55]. Notably, ISRs were reported at rates comparable to gastrointestinal events among tirzepatide users in FAERS [45]. In contrast, an analysis of the EudraVigilance database found higher reporting of ISRs with liraglutide compared to semaglutide and tirzepatide [11]. These differing findings across pharmacovigilance databases likely reflect differences in regional prescribing patterns, duration of market availability, and spontaneous reporting practices rather than true differences in risk.

Among tirzepatide pharmacovigilance reports, injection-site pain was the most frequently reported event, followed by injection-site hemorrhage, erythema, bruising, pruritus, swelling and mass [7,45,46,49]. The spectrum of ISRs reported with semaglutide was broadly similar to that observed with tirzepatide, although bruising accounted for a greater proportion of reports and pruritus was comparatively uncommon. Liraglutide demonstrated a somewhat different profile, with erythema, bruising, and pruritus predominating, whereas injection-site hemorrhage was reported less frequently [7].

Immunogenicity may contribute to the occurrence of ISRs with tirzepatide [25], potentially related to its polypeptide-based structure; however, the relatively low proportion of anti-tirzepatide positive individuals who have ISRs suggests that immunogenicity alone does not account for all ISRs. Incorrect administration is another potential factor that may also contribute, particularly in real-world settings. Compared with semaglutide, tirzepatide has been associated with substantially higher odds (OR—40.13 (36.7–43.8) of incorrect dose administration, particularly the administration of an extra dose [52]—a pattern observed across multiple pharmacovigilance databases, including both FAERS and VigiBase [7,55]. Administration of a greater-than intended dose or volume could plausibly increase local tissue irritation and inflammation, although direct association between dosing errors and ISRs has not been established. In addition, real-world user feedback suggests the tirzepatide autoinjector requires greater plunger force than semaglutide or liraglutide devices, raising the possibility that device-related administration challenges could contribute to improper administration or repeated injection attempts, although this has yet to be directly assessed [7].

### 3.2. Altered Skin Sensation

Altered skin sensation has emerged as a distinctive but incompletely recognized adverse effect of GLP-1 RAs, encompassing dysesthesia, paresthesia, hyperesthesia, allodynia, burning sensation, and skin sensitivity (Table 3). The frequency and recognition of these events has increased with higher-dose formulations typically used for obesity treatment, leading to the addition of dysesthesia as an AE to the Wegovy^®^ (semaglutide) package insert in March 2026 [2]. In clinical trials, altered skin sensation was primarily associated with semaglutide and demonstrates a dose-dependent relationship (Table 3) [26,27,37,56]. Relative risks calculated from aggregated event rates across clinical trials demonstrated a significant association of dysesthesia-related events with both subcutaneous 2.4 mg and 7.2 mg doses, as well as 50 mg oral semaglutide (Table 3). A higher incidence of dysesthesia-related events was observed with the higher dose formulations, both subcutaneous and oral, typically indicated for weight loss [26,27,56,57,58]. Most events were mild, typically presenting as hyperesthesia, sensitive skin, or dysesthesia. Nevertheless, approximately 23% of participants reduced their dose because of sensory symptoms, while temporary or permanent treatment discontinuation occurred in 8% and 2% of participants, respectively [2,26,27].

In contrast, dysesthesia-related events are relatively uncommon for tirzepatide; in the package insert for Mounjaro^®^, dysesthesia was reported across pooled placebo-controlled trials for 5 mg, 10 mg and 15 mg in 0.4% of participants [59]. Altered skin sensations have not been reported with liraglutide.

Pharmacovigilance data provide complementary information on the spectrum of altered skin sensation reported in routine clinical practice. In an analysis of VigiBase, dysesthesia-related events compromised approximately 1% of spontaneous safety reports for incretin-based therapies, with semaglutide accounting for over 30% of these cases followed by tirzepatide (23.6%) and liraglutide (12.3%) [60]. The most common manifestation was paresthesia followed by hypoesthesia and burning sensation. Paresthesia was most often localized in the extremities, whereas burning sensation was spread across the abdomen, back, thighs and arms [60]. The majority of cases were among middle-aged (45–64 years) females, with some cases recording a history of neuropathy [60]. In the Euro Vigilance database, a higher dysesthesia disproportionality signal was found for semaglutide compared to tirzepatide [11]. In disproportionality analysis, tirzepatide also demonstrated significant disproportionality signals for skin sensitization (ROR 9.43 [5.77–15.42]), sensitive skin (ROR: 4.35 [3.56–5.32]) and allodynia (ROR 20.5 [16.59,25.4]), although lower than the signals for semaglutide [51,52]. Case reports in the literature further suggest an association between semaglutide and tirzepatide and symptoms of allodynia and skin burning [61,62,63,64]. Across case reports and cohort studies, dysesthesia appears to occur most often during dose escalation, although onset has been reported from several days to a few months after treatment initiation or dose escalation [2,60,62]. Symptoms may resolve over time without treatment discontinuation, while discontinuation may be warranted in more severe cases. Some patients have been subsequently able to resume treatment following dose interruption and gradual re-escalation [2,60,62].

### 3.3. Hypersensitivity, Rash and Other Non-Serious Cutaneous Adverse Events

Beyond ISRs and altered skin sensation, GLP-1 and GIP/GLP-1 RAs have been associated with a range of additional cutaneous AEs, most commonly rash and hypersensitivity-related reactions (Table 4). Rash and urticaria have been reported in clinical trials for pediatric patients for both semaglutide (3%, *N* = 133) and liraglutide (3.2%, *N* = 125) compared with no reports in placebo groups [3,65]. A similar incidence (3.6%, 28/769) of skin rash or urticaria was reported among adults receiving liraglutide in a retrospective cohort study [66]. In comparison, tirzepatide appears to be less frequently associated with rash. Pharmacovigilance analyses have identified lower reporting of rash with tirzepatide than semaglutide, including a significantly lower disproportionality signal for unspecified rash (ROR 0.42, 95% CI 0.37–0.48) [67]. Similarly, a pharmacovigilance analysis found that rash, pruritus, and hyperhidrosis comprised a lower proportion of spontaneous reports for tirzepatide than semaglutide and liraglutide [68]. Taken together, these findings suggest that the association between GLP-1 and GIP/GLP-1 RAs and rash may be variable, as an analysis of the VigiBase database did not find rash to be meaningfully associated with semaglutide, liraglutide or tirzepatide [48].

Hypersensitivity reactions, including severe cases of urticaria and eczema, have been reported with tirzepatide around 1.9% across trials compared with 0.45% with placebo, although relative risk ratios were not significant (Table 4) [20,21,22]. Delayed hypersensitivity reactions appear to occur more frequently than immediate hypersensitivity reactions [43]. These reactions were slightly more common in those who had developed anti-tirzepatide antibodies (6.2%, 106/2570) compared to those who had not (3%, 73/2455); a pattern observed in pediatric and adult patients [25]. Semaglutide and liraglutide have not reported hypersensitivity reactions consistently across clinical trials. Nevertheless, analysis of pharmacovigilance databases, such as EudraVigilance, has demonstrated low-intensity signals for hypersensitivity reactions with semaglutide and liraglutide as well as angioedema with liraglutide [11].

Data from pharmacovigilance reporting in FAERS and cross-sectional analysis indicate that eczematous, dry skin, rash, pruritus and drug eruptions are among the most common reported cutaneous AEs for GLP-1 RA and GIP/GLP-1 RAs [70,71], particularly for semaglutide and liraglutide [68]. Although one cross-sectional analysis reported skin side effects in 40% of GLP-1 RA or GIP/GLP-1 RA users [71], these findings should be interpreted cautiously given the potential for reporting and ascertainment bias and the absence of appropriate comparators. Indeed, an analysis of the TriNetX database found lower rates of pruritus among diabetic users of semaglutide, liraglutide, and tirzepatide compared with controls, suggesting that these agents may have beneficial effects on pruritus in some populations [72]. Collectively, these findings highlight the importance of appropriate active comparators and controlled study designs to distinguish drug-related cutaneous reactions from underlying disease, comorbidities, and other factors influencing skin symptoms.

### 3.4. Uncommon Cutaneous Adverse Events

As the use of GLP-1 and GIP/GLP-1 RA therapies expands, an increasing number of uncommon cutaneous adverse reactions have been reported in association with drug use. These reactions can be broadly categorized into autoimmune and eosinophilic disorders, angioedema and severe allergic reactions with skin manifestations, and vasculitis or other severe cutaneous adverse reactions (Table 5). Although uncommon, these events are clinically important because they typically require drug discontinuation to resolve.

Autoimmune blistering and eosinophilic disorders are uncommonly reported immune-mediated reactions in GLP-1 RA and GIP/GLP-1 RAs users. In a large real-world study emulating a clinical trial using the TriNetX US Collaborative Network, GLP-1 RAs were associated with a lower risk of bullous pemphigoid and pemphigus vulgaris compared with dipeptidyl peptidase 4 inhibitors (DPP4is) [73]. Further, in a large retrospective cohort study of veterans from 2006 to 2021, GLP-1 RA use was not associated with an increased risk of developing bullous pemphigoid or pemphigus vulgaris [74]. That being said, several case reports have described bullous pemphigoid or pemphigus vulgaris in association with GLP-1 RA use and an improvement after drug discontinuation raising the possibility of a drug-related effect in individual cases [75,76,77,78,79]. Presentations of autoimmune blistering disorders or eosinophilic-associated dermatoses may initially be mistaken for erythema multiforme or nonspecific drug eruptions, highlighting the importance of maintaining clinical suspicion in patients presenting with new blistering or eosinophilic skin disease after GLP-1 RA initiation [78]. Severe cutaneous adverse reactions have also been identified in pharmacovigilance analyses of FAERS, majorly in females, with a median age of 63 years [67].

**Table 5:** Rare cutaneous reactions associated with GLP-1 and GIP/GLP-1 RAs in case series/reports.

Drug	Diagnosis	Evidence Type	Diagnostic Confirmation	Age/Sex	Indication	Latency	Dechallenge/Rechallenge *	Other Comorbidities/Medical History	Ref.
Eosinophilic-related or autoimmune blistering conditions									
Semaglutide	Bullous pemphigoid	Case report	Yes—H/E, IF	61/F	T2DM	~1 month	+/N	HTN, HLD, lichen sclerosus, fibromyalgia, chronic gingivitis, RA	[76]
Semaglutide	Bullous pemphigoid	Case report	Yes—Histo	68/F	T2DM	4 months	−/N	HTN, renal disease	[75]
Liraglutide	Bullous pemphigoid	Case report	Yes – Histo	75/M	T2DM	2 wks	−/N	HTN, stroke, prostate cancer	[77]
Tirzepatide	Bullous pemphigoid	Case report	Yes—Histo	46/M	T2DM	Shortly after	+T/N	CKD, positive antinuclear antibodies, SSA antibodies	[78]
Semaglutide	Eosinophil fasciitis	Case report	No—biopsy	42/F	WM	2 wks	+T/N	None	[80]
Tirzepatide	Lichen planus pigmentosus	Case report	Yes—biopsy	46/F	WM	~6 months	N/N	None	[81]
Semaglutide	Drug reaction with eosinophilia (2)	Pharmacovigilance	NR	NR	NR	NR	NR	NR	[67]
Tirzepatide	Drug reaction with eosinophilia	Pharmacovigilance	NR	NR	NR	NR	NR	NR	[67]
Drug eruptions & reactions									
Semaglutide	Fixed drug eruption	Case report	Yes—biopsy	62/F	T1DM, WM	72 h	+/N	HTN	[82]
Semaglutide	Granuloma annulare	Case report	Yes—Histo	73/F	T2DM/WM	2 wks after dose esc.	+/N, tolerated tirzepatide		[83]
Semaglutide	Photodistrubuted exfoliative dermatitis	Case report	Yes—Histo	77/M	WM	2 months around dose esc.	+/N	Carcinoma, keratoacanthoma, HTN, COPD, HLD, venous thromboembolism	[84]
Semaglutide	Generalized exanthematous pustulosis^p^	Pharmacovigilance	NR	NR	NR	NR	NR	NR	[67]
Semaglutide (compounded)	SDRIFE	Case report	Yes	25/F	NR	Delayed, but 2 days after compounded dose	+T/N	NR	[85]
Semaglutide	Dermal hypersensitivity (2)	Case series	Yes	75/F 74/M	T2DM	10 months 1 month	+/N +/N	NR	[86]
Semaglutide	Maculopapular exanthema	Case report	NR	34/M	WM	Dose esc.	N/N Subcut. desensitization	NR	[87]
Liraglutide	Delayed hypersensitivity	Case report	Yes—delayed skin test positive	56/F	WM	1 month after dose esc.	+T/N—semaglutide successful	Prediabetes, HTN, HTG, AR, gastroesophageal reflux disease	[88]
Liraglutide	Delayed hypersensitivity	Case report	Yes—delayed skin test positive	42/F	WM	1 wk after dose esc.	+/N	None	[89]
Liraglutide	APEP	Case report	Yes	40/F	T2DM	1–2 days	+T/N	Autoimmune disorders, psoriasis, arthralgias, oral ulcers	[90]
Liraglutide	Drug reaction (psoriasiform drug rash)	Case report	Yes—biopsy	68/M	T2DM	2.5 wks	−/N	CKD, leg ulcers	[91]
Liraglutide	Eczematous, prurigo-like eruption	Case report	No	56/M	WM	2 months	−/N	AR, conjunctivitis	[92]
Liraglutide	Edema and necrotic ulcers	Case report	Yes	44/M	WM	10 days	N/N	None	[93]
Tirzepatide	SDRIFE	Case report	No	37/F	NR	3 wks	+/N	None	[94]
Semaglutide	Stevens-Johnson syndrome (2)	Pharmacovigilance	NR	NR	NR	NR	NR	NR	[67]
Tirzepatide	Stevens-Johnson syndrome (3)	Pharmacovigilance	NR	NR	NR	NR	NR	NR	[67]
Angioedema/anaphylaxis									
Semaglutide (compounded)	Angioedema	Case report	No	50/F	WM	4 h	N/N	HTN, lisinopril-induced angioedema	[95]
Semaglutide	Angioedema and anaphylaxis	Case report	No	59/F	NR	1 month, occurred 2 days after injection	+/N	NR	[96]
Semaglutide	Anaphylaxis with hives and angioedema	Case report	No	NR	NR	15 min	N/N Subcut. liraglutide tolerated	NR	[97]
Tirzepatide	Biphasic anaphylaxis	Case report	No	67/M	T2DM	20 min	N/N	HTN, HLD, obstructive sleep apnea	[98]
Tirzepatide	Hypersensitivity	Case report	Yes—skin testing	29/F	WM	15 h after 3rd dose of 7.5 mg	+/N, tolerated semaglutide	HTN	[99]
Tirzepatide	Allergic reaction- urticaria rash	Case report	No	67/F	T2DM	10–15 min	+/N	Obesity, HTN, HChol	[100]
Liraglutide	Angioedema	Case report	No	58/M	T2DM	6 h.	+T/N	NR	[101]
Other									
Semaglutide	Leukocytoclastic vasculitis	Case report	Yes—Histo	40/M	WM	~8 months, 4 wks. after dose esc.	+/N	HTN, gastroesophageal reflux	[102]
Semaglutide	Leukocytoclastic vasculitis	Case report	Yes—Histo	73/M	T2DM	2 wks.	+/N	HTN, HChol, diabetic peripheral neuropathy, chronic venous insufficiency, hypogonadism	[103]
Semaglutide	Leukocytoclastic vasculitis	Case report	Yes	65/F	WM	2 wks.	+/N	HTN, HLD	[104]
Liraglutide	Vesiculopustular dermatosis	Case report	Yes—Histo	59/M	T2DM/WM	6 wks.	+/N	Renal stones, percutaneous coronary intervention	[105]
Tirzepatide	Necrotizing soft tissue disorder	Case report	NR	43/F	T2DM/WM	NR	N/N	None	[106]
Tirzepatide	Guttate psoriasis	Case report	NR	59/F	WM	7 months	−/N	None	[107]

* +, positive dechallenge; +T, positive dechallenge was combined with treatment; −, negative dechallenge; N, no dechallenge; APEP, acute photodistributed exanthematous pustulosis; AR, allergic rhinitis; CKD, chronic kidney disease; COPD, chronic obstructive pulmonary disorder; HChol, hypercholesterolemia; H/E, hematoxylin and eosin; Histo, histopathology; HLD, hyperlipidemia; HTG, hypertriglyceridemia; HTN, hypertension; IF, immunofluorescence; NR, not reported; SDRIFE, symmetrical drug-related intertriginous and flexural exanthema; Subcut, subcutaneous; RA, rheumatoid arthritis; T2DM, type 2 diabetes mellitus; WM, weight management.

Observational evidence regarding melanoma and skin cancer is inconsistent. A retrospective propensity-matched cohort study in TriNetX network found a higher risk of melanoma among individuals who had initiated liraglutide for T2DM within the past 5 years compared with various active comparators (e.g., DPP4i, SGLT2i), whereas semaglutide was not associated with skin cancer [108]. Conversely, a TriNetX propensity score-matched analysis of a large cohort of individuals with obesity found a lower risk of skin cancer among users of GLP-1 RAs, including liraglutide, semaglutide, and dulaglutide, compared with matched controls [109]. However, the latter analysis evaluated GLP-1 RAs collectively and did not provide drug-specific estimates, limiting conclusions regarding liraglutide. Given these conflicting observational findings and differences in study populations, the relationship between individual GLP-1 RA use and melanoma remains uncertain.

One potential mechanism underlying the apparent association, albeit rare, of GLP-1 and GIP/GLP-1 RAs with several immune-mediated conditions may be related to the presence of GLP-1 receptors on various immune cells and the impact of GLP-1 RAs on immune processes. For example, GLP-1 receptors are expressed on human eosinophils and neutrophils, and previous research indicates that GLP-1 RAs may regulate eosinophil activation and neutrophil migration [104,110]. This may be particularly relevant for leukocytoclastic vasculitis where vascular injury is driven by neutrophil infiltration and the subsequent release of proteolytic enzymes [104]; however, more research is needed to understand the potential mechanisms underlying these associations. Given these conditions are very uncommon among the population, cases should be approached on an individualized basis.

## 4. Discussion of Clinical Implications

Cutaneous AEs are generally mild and self-limiting, more often reported in females and older individuals [12]. Pediatric patients also may be more susceptible to rash and urticaria reactions with semaglutide and liraglutide injections than adults, although more research is needed in this patient population [111]. Most reported cutaneous reactions develop within the first several weeks after treatment initiation around dose escalation, although delayed presentations occurring months later have also been described (Table 5) [51,81,107,112]. In many cases, reactions are transient and resolve spontaneously [113,114]. Management should be guided by the severity and clinical characteristics of the reaction. Mild ISRs may be managed with local measures, whereas persistent or bothersome reactions may warrant treatment modification. In such cases, successfully switching to an alternative GLP-1 RA or dual GIP/GLP-1 RAs has been described, and may be considered based on individual clinical context [99,115]. Switching to an agent with a lower reported frequency of ISRs, such as semaglutide, or to oral semaglutide when clinically appropriate, may be possible. For suspected immediate hypersensitivity, discontinuation of the drug and an appropriate allergy evaluation should be considered. Antihistamines and clobetasol propionate ointment have been used in some reported cases and may have a role in symptom management [116,117], although evidence regarding their ability to permit continued treatment is limited. According to guidance from the American Academy of Allergy, Asthma, and Immunology, continuation of the suspected medication with concomitant antihistamine therapy (or corticosteroids) may be considered for non-systemic, T-cell mediated cutaneous reactions, whereas Ig-E mediated reactions or more severe T-cell-mediated reactions generally warrant discontinuation [118,119]. For patients with cutaneous reactions who require continued therapy, subcutaneous stock desensitization has also been reported [87]. In more severe or prolonged cases, drug discontinuation alone is frequently sufficient for clinical improvement [103,120]. Severe drug eruptions, vasculitis or eosinophilic reactions warrant discontinuation of the suspected drug and specialist assessment.

Clinically, distinguishing direct drug-related cutaneous reactions from weight-loss-associated dermatologic changes is important when interpreting the safety profile of GLP-1 and GIP/GLP-1 RAs. The present review identified predominantly mild cutaneous reactions that may require treatment modification in some cases. Conversely, facial volume loss, hollowness, skin laxity, and telogen effluvium are more plausibly related to substantial or rapid weight loss and may be managed without discontinuing therapy. This distinction may help clinicians contextualize dermatologic complaints and avoid attributing all treatment-associated skin changes directly to the drug.

## 5. Limitations

Several important limitations should be considered. Only clinical trials that reported or coded AEs of interest were included, and AE ascertainment was not consistent across trials. Further, although clinical trials were additionally sought and screened for relevant AEs, some clinical trials may not have been identified through the search strategy if AEs of interest were not reported in the title or abstract. Pharmacovigilance data, case series, and case reports lack standardized denominators and, in some cases, appropriate comparators, limiting estimates of incidence and causality. Pharmacovigilance studies overlapping in time periods may result in duplicate reporting and these analyses should not be interpreted as independent confirmations. The majority of rare events depended upon spontaneous reports and case reports, which are additionally susceptible to reporting bias and confounding. Aggregate RRs were calculated from combined event counts rather than through formal meta-analysis, these estimates do not account for differences in study size or between-study heterogeneity and should be interpreted descriptively. Finally, screening was conducted by a single reviewer and the search was restricted to English-language publications.

## 6. Conclusions

Overall, GLP-1 and GIP/GLP-1 RAs demonstrate a generally favorable cutaneous safety profile, with most AEs being mild and manageable. ISRs and altered skin sensation represent the best-supported cutaneous AEs, with ISRs reported more frequently with tirzepatide and altered skin sensation reported most prominently with semaglutide, particularly at higher doses. Rash and hypersensitivity-related reactions are also reported, including in clinical trials and pharmacovigilance datasets; however, findings are inconsistent across studies, and current evidence does not establish clear drug-specific risks. Less frequent drug eruptions and immune-mediated reactions, particularly those described in case reports and spontaneous reports, should be interpreted as potential safety signals rather than established drug-specific risks. Continued pharmacovigilance and systematic reporting of cutaneous AEs in clinical trials, alongside appropriate comparator groups, will be important to clarify their incidence, risk factors, and potential mechanisms and to support individualized monitoring and management.

## Figures and Tables

**Table 2:** Pharmacovigilance signals for injection-site (IS) reactions with GLP-1 RA-based therapies in FAERS.

	Reporting Odds Ratio		
FAERS *	Semaglutide (ROR 95%Cl) [7]	Liraglutide (ROR 95%Cl) [7]	Tirzepatide (Range of Reported ROR Across FAERS) [7,46,47,49,50,51,52,53]
IS pain	0.91 (0.8–1.04)	0.66 (0.55–0.8)	8.35–18.46
IS hemorrhage	1.58 (1.22–1.87)	0.82 (0.61–1.1)	13.17–30.49
IS erythema	0.66 (0.51–0.86)	1.86 (1.58–2.2)	2.76–18.65
IS bruising	1.68 (1.38–2.04)	2.49 (2.09–2.98)	3.72–14.98
IS pruritus	0.17 (0.09–0.33)	2.66 (2.22–3.2)	2.27–16.13
IS mass	1.5 (1.17–1.93)	2.05 (1.57–2.68)	6.48–13.31
IS rash	0.16 (0.06–0.42)	2.99 (2.3–3.89)	2.34–15.43
IS swelling	0.61 (0.45–0.84)	0.61 (0.43–0.88)	3.22–6.32
IS urticaria	-	-	4.33–16.52
IS warmth	-	-	4.13–8.07
IS coldness	-	-	45.06–148.7
IS discoloration	-	-	2.61–4.69
IS paresthesia	-	-	13.32–37.84
IS hypersensitivity	-	-	6.29–13.75
IS vesicles	-	-	3.53–9.14
IS dermatitis	-	-	21.69

FAERS, Food and Drug Administration Adverse Events Reporting System; IS, injection site; ROR, reporting odds ratio. * Injection-site reactions are ordered from top to bottom based on number of reports for each adverse event reported in Guo et al., 2026 [52] and Zhang et al., 2025 [49] for tirzepatide which comprehensively cover all of the types of injection-site reactions over covers the longest duration of time. These metrics compared the frequency of specific adverse events reported for the GLP-1 RA or GIP/GLP-1 RA to all other drugs in the FAERS database. Included studies in FAERS had the following date range for reporting: Chen et al., 2025 [47] Q2 2022–Q4 2023; Li et al., 2025 [50] Q2 2022–Q4 2023; Liu et al., 2024 [46] Q1 2022–Q3 2023; Ou et al., 2024 [53] Q2 2022–Q1 2024; Hindi et al., 2026 [7] Q from approval semaglutide 2018, liraglutide 2013, tirzepatide Q1 2023—Jun 2024; Guo et al., 2026 [52] Q2 2022—Q4 2025; Gu et al., 2026 [51] Q2 2022–Q1 2025; Zhang et al., 2025 [49] May 2022–2024. Included studies have overlapping date ranges for analysis of the same database and should not be interpreted as independent reports of these adverse events. It should be noted that these adverse events were reported at much lower frequency than the reporting odds for the control group.

**Table 3:** Altered skin sensation associated with GLP-1 RA-based therapies in RCTs.

GLP-1 or GIP/GLP-1 RA	GLP-1 or GIP/GLP-1 RA Dose (Patients with Adverse Event/Total Taking Medication)				Control
**Semaglutide—Oral**	**25 mg/d [58]**		**50 mg/d [56,57]**		**Placebo**
	10/204 [4.9%] RR: 10.0 [0.59,170.35]		56/468 [12.0%] RR: **11.6 [4.72, 28.47]**		5/501 [1.0%]
**Semaglutide—Subcutaneous** [26,27]	**2.4 mg/wk.**		**7.2 mg/wk.**		**Placebo**
	17/304 [5.6%] RR: **16.9 [2.27, 126.53]**		288/1311 [22%] RR: **66.6 [9.38, 472.29]**		1/303 [0.33%]
Tirzepatide *	**5 mg**	**10 mg**		**15 mg**	**Placebo**
Mounjaro [59]	0.4%	0.4%		0.4%	0%
Zepbound [4]	0.2%	0.2%		0.4%	0.1%
**Liraglutide**	N/A				

Cases included any dysesthesia-related symptoms including allodynia, burning sensation, hyperesthesia, pain of skin, paresthesia, sensitive skin, skin burning sensation, skin discomfort, and skin sensitization. Relative risk calculations are presented as relative risk (95% confidence interval). Relative risks where the 95% confidence interval excludes 1.0 were considered statistically significant and bolded. * Information from pooled clinical trials in package insert. GIP, glucose-dependent insulinotropic polypeptide; GLP-1 RA, Glucagon-like peptide-1 receptor agonist; RR, relative risks.

**Table 4:** Hypersensitivity, rash and urticaria associated with GLP-1 RA-based therapies in RCTs.

	Semaglutide		Liraglutide				Tirzepatide
Rash/urticaria	**2.4 mg** [2]		**1.8 mg** [69]		**3 mg** [3,69]		N/A
	4/133 [3%] vs. 0/67 [0%] placebo ^a^		7/90 [7.8%] vs. 2/98 [2.0%] placebo		1/93 [1.1%] vs. 2/98 [2.0%] placebo 4/125 [3.2%] vs. 0/126 [0%] placebo ^a^		
	**Semaglutide** [9]	**Liraglutide**	**Tirzepatide** [9,20,21,22,24,43]				**Placebo** [20,21,22]
Hypersensitivity	**1 mg**	N/A	**5 mg**	**10 mg**		**15 mg**	4/876 [0.45%]
	11/469 [2.3%]		35/1843 [1.9%] 11/867 [1.3%] RR_PL_: 2.8 [0.9, 8.7] 10/358 [2.8%] RR_IN_: 2.0 [0.7, 5.8]	35/1852 [1.8%] 7/876 [0.8%] RR_PL_:1.7 [0.5, 5.9] 12/360 [3.3%] RR_IN_: 2.4 [0.8, 6.7]		33/1846 [1.8%] 8/870 [0.9%] RR_PL_: 2.0 [0.6, 6.7] 8/358 [2.5%] RR_IN_: 1.8 [0.6, 5.3]	**Insulin degluc** [24]
							5/359 [1.4%]

Incidence numbers out of total were reported across all clinical trials and reported as the crude incidence (values underlined); however, only clinical trials with placebo or insulin control groups were included in the relative risk calculations. Incidences corresponding to the relative risk calculations are included directly above the relative risk. Relative risk calculations are presented as relative risk (95% confidence interval). Relative risks where the confidence interval excludes 1.0 are considered statistically significant and bolded. RR_PL_, relative risk compared to placebo; RR_IN_, relative risk compared to insulin-degludec or insulin glargine. a—Study was conducted in pediatrics or adolescents.

## Data Availability

No new data were generated in this study. Relative risk calculations from clinical trial data were calculated from incidence values reported in the cited references.

## Supplementary Materials

The following supporting information can be downloaded at: https://www.mdpi.com/article/10.3390/medsci14060652/s1, Figure S1: Risk of bias for randomized controlled clinical trials. Figure S2: ROBINS-E for non-randomized observational studies. Table S1: JBI Critical Appraisal Checklist for Case Reports. File S1: PRISMA checklist.

## Abbreviations

The following abbreviations are used in this manuscript:

AE	Adverse event
DPP4i	Dipeptidyl peptidase-4 inhibitor
CI	Confidence interval
FAERS	Food and Drug Administration Adverse Events Reporting System
ISR	Injection-site reaction
GIP	Glucose-dependent insulinotropic polypeptide
GLP-1 RA	Glucagon-like peptide 1 receptor agonist
RCT	Randomized controlled trial
RR	Relative risk
ROR	Reporting odds ratio
T2DM	Type 2 diabetes
SGLT2i	Sodium-glucose cotransporter-2 inhibitor

## References

[1] Rosen C.J., Ingelfinger J.R. (2026). GLP-1 Receptor Agonists. N. Engl. J. Med..

[2] Novo Nordisk Inc (2026). Wegovy Highlights of Prescribing Information.

[3] Novo Nordisk Inc (2026). Saxenda Highlights of Prescribing Information.

[4] Eli Lily and Company (2026). Zepbound Highlights of Prescribing Information.

[5] Gratzl S., Farrar K.G., Holler E., Vachon M.S., Masters N., Cartwright B.M.G. (2026). Monitoring Report: GLP-1 RA Prescribing Trends–March 2026 Data. medRxiv.

[6] de Carvalho T.R., Matzenbacher L.S., de Barros L.G.B., Blank L.M., Perrone M.K., Haussen A.G.R., Rizzolli J., Telo G.H. (2026). Self-Medication with GLP-1 Receptor Agonists among Brazilian Adults: Findings from a Web-Based Survey. Int. J. Obes..

[7] Hindi A., Mekkawy M., Shokr H. (2026). Psychiatric Adverse Events and Administration Challenges Associated with GLP-1 Receptor Agonists for Weight Loss: A Real-World Analysis. Pharmaceuticals.

[8] Ismaiel A., Scarlata G.G.M., Boitos I., Leucuta D.C., Popa S.L., Al Srouji N., Abenavoli L., Dumitrascu D.L. (2025). Gastrointestinal Adverse Events Associated with GLP-1 RA in Non-Diabetic Patients with Overweight or Obesity: A Systematic Review and Network Meta-Analysis. Int. J. Obes..

[9] Frías J.P., Davies M.J., Rosenstock J., Pérez Manghi F.C., Fernández Landó L., Bergman B.K., Liu B., Cui X., Brown K. (2021). Tirzepatide versus Semaglutide Once Weekly in Patients with Type 2 Diabetes. N. Engl. J. Med..

[10] Aronne L.J., Horn D.B., le Roux C.W., Ho W., Falcon B.L., Gomez Valderas E., Das S., Lee C.J., Glass L.C., Senyucel C. (2025). Tirzepatide as Compared with Semaglutide for the Treatment of Obesity. N. Engl. J. Med..

[11] Popa Ilie I.R., Ghibu S., Butuca A., Dobrea C.M., Frum A., Homorodean C., Chis A.A., Morgovan C. (2026). Incretin-Based Drugs for Obesity: Common and Drug-Specific Reporting Patterns of Adverse Drug Reactions—A Comparative Disproportionality Analysis Using EudraVigilance Reports Integrating SmPC Data. Pharmaceuticals.

[12] Fat M.N., Johnson H.C., Farberg A.S. (2026). Cutaneous Adverse Events Associated With GLP-1 Receptor Agonists: A FAERS Database Analysis From 2018-2024. J. Drugs Dermatol..

[13] Barone M., Brunetti B., D’Emilio R., Caputo M.G., Tenna S., Persichetti P. (2026). Effects of GLP-1 Receptor Agonists on Skin Quality: A Comprehensive Literature Review. Aesthetic Plast. Surg..

[14] Cheng P.L., Chang H.C. (2026). Glucagon-like Peptide-1 Receptor Agonists and Hair Loss: A Systematic Review and Meta-Analysis. Diabetes Res. Clin. Pract..

[15] Sterne J.A.C., Savović J., Page M.J., Elbers R.G., Blencowe N.S., Boutron I., Cates C.J., Cheng H.-Y., Corbett M.S., Eldridge S.M. (2019). RoB 2: A Revised Tool for Assessing Risk of Bias in Randomised Trials. BMJ.

[16] Higgins J.P.T., Morgan R.L., Rooney A.A., Taylor K.W., Thayer K.A., Silva R.A., Lemeris C., Akl E.A., Bateson T.F., Berkman N.D. (2024). A Tool to Assess Risk of Bias in Non-Randomized Follow-up Studies of Exposure Effects (ROBINS-E). Environ. Int..

[17] Moola S., Munn Z., Tufanaru C., Aromataris E., Sears K., Sfetcu R., Currie M., Qureshi R., Mattis P., Lisy K., Aromataris E., Munn Z. (2020). Systematic Reviews of Etiology and Risk. JBI Manual for Evidence Synthesis.

[18] Mishra R., Raj R., Elshimy G., Zapata I., Kannan L., Majety P., Edem D., Correa R. (2023). Adverse Events Related to Tirzepatide. J. Endocr. Soc..

[19] Wadden T.A., Chao A.M., Machineni S., Kushner R., Ard J., Srivastava G., Halpern B., Zhang S., Chen J., Bunck M.C. (2023). Tirzepatide after Intensive Lifestyle Intervention in Adults with Overweight or Obesity: The SURMOUNT-3 Phase 3 Trial. Nat. Med..

[20] Jastreboff A.M., Aronne L.J., Ahmad N.N., Wharton S., Connery L., Alves B., Kiyosue A., Zhang S., Liu B., Bunck M.C. (2022). Tirzepatide Once Weekly for the Treatment of Obesity. N. Engl. J. Med..

[21] Dahl D., Onishi Y., Norwood P., Huh R., Bray R., Patel H., Rodríguez Á. (2022). Effect of Subcutaneous Tirzepatide vs Placebo Added to Titrated Insulin Glargine on Glycemic Control in Patients With Type 2 Diabetes. JAMA.

[22] Rosenstock J., Wysham C., Frías J.P., Kaneko S., Lee C.J., Fernández Landó L., Mao H., Cui X., Karanikas C.A., Thieu V.T. (2021). Efficacy and Safety of a Novel Dual GIP and GLP-1 Receptor Agonist Tirzepatide in Patients with Type 2 Diabetes (SURPASS-1): A Double-Blind, Randomised, Phase 3 Trial. Lancet.

[23] Del Prato S., Kahn S.E., Pavo I., Weerakkody G.J., Yang Z., Doupis J., Aizenberg D., Wynne A.G., Riesmeyer J.S., Heine R.J. (2021). Tirzepatide versus Insulin Glargine in Type 2 Diabetes and Increased Cardiovascular Risk (SURPASS-4): A Randomised, Open-Label, Parallel-Group, Multicentre, Phase 3 Trial. Lancet.

[24] Ludvik B., Giorgino F., Jódar E., Frias J.P., Fernández Landó L., Brown K., Bray R., Rodríguez Á. (2021). Once-Weekly Tirzepatide versus Once-Daily Insulin Degludec as Add-on to Metformin with or without SGLT2 Inhibitors in Patients with Type 2 Diabetes (SURPASS-3): A Randomised, Open-Label, Parallel-Group, Phase 3 Trial. Lancet.

[25] Mullins G.R., Hodsdon M.E., Li Y.G., Anglin G., Urva S., Schneck K., Bardos J.N., Martins R.F., Brown K., Calderon B. (2024). Tirzepatide Immunogenicity on Pharmacokinetics, Efficacy, and Safety: Analysis of Data From Phase 3 Studies. J. Clin. Endocrinol. Metabol..

[26] Lingvay I., Bergenheim S.J., Buse J.B., Freitas P., Garvey W.T., Harder-Lauridsen N.M., Rosenstock J., Sahu K., Wharton S. (2025). Once-Weekly Semaglutide 7·2 Mg in Adults with Obesity and Type 2 Diabetes (STEP UP T2D): A Randomised, Controlled, Phase 3b Trial. Lancet Diabetes Endocrinol..

[27] Wharton S., Freitas P., Hjelmesæth J., Kabisch M., Kandler K., Lingvay I., Quiroga M., Rosenstock J., Garvey W.T. (2025). Once-Weekly Semaglutide 7·2 Mg in Adults with Obesity (STEP UP): A Randomised, Controlled, Phase 3b Trial. Lancet Diabetes Endocrinol..

[28] Elkind-Hirsch K.E., Chappell N., Shaler D., Storment J., Bellanger D. (2022). Liraglutide 3 Mg on Weight, Body Composition, and Hormonal and Metabolic Parameters in Women with Obesity and Polycystic Ovary Syndrome: A Randomized Placebo-Controlled-Phase 3 Study. Fertil. Steril..

[29] Danne T., Biester T., Kapitzke K., Jacobsen S.H., Jacobsen L.V., Carlsson Petri K.C.C., Hale P.M., Kordonouri O. (2017). Liraglutide in an Adolescent Population with Obesity: A Randomized, Double-Blind, Placebo-Controlled 5-Week Trial to Assess Safety, Tolerability, and Pharmacokinetics of Liraglutide in Adolescents Aged 12-17 Years. J. Pediatr..

[30] Rubino D.M., Greenway F.L., Khalid U., O’Neil P.M., Rosenstock J., Sørrig R., Wadden T.A., Wizert A., Garvey W.T., Arauz-Pacheco C. (2022). Effect of Weekly Subcutaneous Semaglutide vs Daily Liraglutide on Body Weight in Adults With Overweight or Obesity Without Diabetes. JAMA.

[31] Neeland I.J., Marso S.P., Ayers C.R., Lewis B., Oslica R., Francis W., Rodder S., Pandey A., Joshi P.H. (2021). Effects of Liraglutide on Visceral and Ectopic Fat in Adults with Overweight and Obesity at High Cardiovascular Risk: A Randomised, Double-Blind, Placebo-Controlled, Clinical Trial. Lancet Diabetes Endocrinol..

[32] Lundgren J.R., Janus C., Jensen S.B.K., Juhl C.R., Olsen L.M., Christensen R.M., Svane M.S., Bandholm T., Bojsen-Møller K.N., Blond M.B. (2021). Healthy Weight Loss Maintenance with Exercise, Liraglutide, or Both Combined. N. Engl. J. Med..

[33] Wadden T.A., Hollander P., Klein S., Niswender K., Woo V., Hale P.M., Aronne L., NN8022-1923 Investigators (2013). Weight Maintenance and Additional Weight Loss with Liraglutide after Low-Calorie-Diet-Induced Weight Loss: The SCALE Maintenance Randomized Study. Int. J. Obes..

[34] Pi-Sunyer X., Astrup A., Fujioka K., Greenway F., Halpern A., Krempf M., Lau D.C.W., le Roux C.W., Violante Ortiz R., Jensen C.B. (2015). A Randomized, Controlled Trial of 3.0 Mg of Liraglutide in Weight Management. N. Engl. J. Med..

[35] Mastrandrea L.D., Witten L., Carlsson Petri K.C., Hale P.M., Hedman H.K., Riesenberg R.A. (2019). Liraglutide Effects in a Paediatric (7-11 y) Population with Obesity: A Randomized, Double-blind, Placebo-controlled, Short-term Trial to Assess Safety, Tolerability, Pharmacokinetics, and Pharmacodynamics. Pediatr. Obes..

[36] Garvey W.T., Batterham R.L., Bhatta M., Buscemi S., Christensen L.N., Frias J.P., Jódar E., Kandler K., Rigas G., Wadden T.A. (2022). Two-Year Effects of Semaglutide in Adults with Overweight or Obesity: The STEP 5 Trial. Nat. Med..

[37] Wadden T.A., Bailey T.S., Billings L.K., Davies M., Frias J.P., Koroleva A., Lingvay I., O’Neil P.M., Rubino D.M., Skovgaard D. (2021). Effect of Subcutaneous Semaglutide vs Placebo as an Adjunct to Intensive Behavioral Therapy on Body Weight in Adults with Overweight or Obesity: The STEP 3 Randomized Clinical Trial. JAMA.

[38] Wilding J.P.H., Batterham R.L., Calanna S., Davies M., Van Gaal L.F., Lingvay I., McGowan B.M., Rosenstock J., Tran M.T.D., Wadden T.A. (2021). Once-Weekly Semaglutide in Adults with Overweight or Obesity. N. Engl. J. Med..

[39] Kadowaki T., Isendahl J., Khalid U., Lee S.Y., Nishida T., Ogawa W., Tobe K., Yamauchi T., Lim S. (2022). Semaglutide Once a Week in Adults with Overweight or Obesity, with or without Type 2 Diabetes in an East Asian Population (STEP 6): A Randomised, Double-Blind, Double-Dummy, Placebo-Controlled, Phase 3a Trial. Lancet Diabetes Endocrinol..

[40] Rubino D., Abrahamsson N., Davies M., Hesse D., Greenway F.L., Jensen C., Lingvay I., Mosenzon O., Rosenstock J., Rubio M.A. (2021). Effect of Continued Weekly Subcutaneous Semaglutide vs Placebo on Weight Loss Maintenance in Adults With Overweight or Obesity. JAMA.

[41] Mu Y., Bao X., Eliaschewitz F.G., Hansen M.R., Kim B.T., Koroleva A., Ma R.C.W., Yang T., Zu N., Liu M. (2024). Efficacy and Safety of Once Weekly Semaglutide 2·4 Mg for Weight Management in a Predominantly East Asian Population with Overweight or Obesity (STEP 7): A Double-Blind, Multicentre, Randomised Controlled Trial. Lancet Diabetes Endocrinol..

[42] Inagaki N., Takeuchi M., Oura T., Imaoka T., Seino Y. (2022). Efficacy and Safety of Tirzepatide Monotherapy Compared with Dulaglutide in Japanese Patients with Type 2 Diabetes (SURPASS J-Mono): A Double-Blind, Multicentre, Randomised, Phase 3 Trial. Lancet Diabetes Endocrinol..

[43] Kadowaki T., Chin R., Ozeki A., Imaoka T., Ogawa Y. (2022). Safety and Efficacy of Tirzepatide as an Add-on to Single Oral Antihyperglycaemic Medication in Patients with Type 2 Diabetes in Japan (SURPASS J-Combo): A Multicentre, Randomised, Open-Label, Parallel-Group, Phase 3 Trial. Lancet Diabetes Endocrinol..

[44] Davies M.J., Bergenstal R., Bode B., Kushner R.F., Lewin A., Skjøth T.V., Andreasen A.H., Jensen C.B., DeFronzo R.A. (2015). Efficacy of Liraglutide for Weight Loss Among Patients With Type 2 Diabetes. JAMA.

[45] Almansour H.A., Thaibah H.A., Alfarhan M., Al-Qahtani S.A., Khardali A.A., Alshammari T.M. (2025). Real-World Safety Concerns of Tirzepatide: A Retrospective Analysis of FAERS Data (2022–2025). Healthcare.

[46] Liu L. (2024). A Real-World Data Analysis of Tirzepatide in the FDA Adverse Event Reporting System (FAERS) Database. Front. Pharmacol..

[47] Chen H., Ding Y., Shan Y. (2025). Post-Marketing Safety Monitoring of Tirzepatide: A Pharmacovigilance Study Based on the FAERS Database. Expert Opin. Drug Saf..

[48] Kim T.H., Lee K., Park S., Oh J., Park J., Jo H., Lee H., Cho J., Wen X., Cho H. (2025). Adverse Drug Reaction Patterns of GLP-1 Receptor Agonists Approved for Obesity Treatment: Disproportionality Analysis from Global Pharmacovigilance Database. Diabetes Obes. Metab..

[49] Zhang Z., Li J., Zheng J., Liang Y., Ma L., Su L. (2025). Tirzepatide Safety in Type 2 Diabetes: A Disproportionality Analysis of Adverse Events Using the FDA FAERS Database. Endocr. Connect..

[50] Li J., Xie J., Han Y., Zhang W., Wang Y., Jiang Z. (2025). A Real-World Disproportionality Analysis of Tirzepatide-Related Adverse Events Based on the FDA Adverse Event Reporting System (FAERS) Database. Endocr. J..

[51] Gu S. (2026). Adverse Events Associated with Tirzepatide: Updated Pharmacovigilance Analysis Using FAERS (2022 Q1-2025 Q1) with an Adapted Time-to-Onset Method. Drug Healthc. Patient Saf..

[52] Guo X., Zhang J., Li Q., Ye Z., Yuan S. (2026). Safety Profile of Tirzepatide in Real-World Clinical Practice: A Pharmacovigilance Study Using the FAERS Database. Diabetes Obes. Metab..

[53] Ou Y., Cui Z., Lou S., Zhu C., Chen J., Zhou L., Zhao R., Wang L., Zou F. (2024). Analysis of Tirzepatide in the US FDA Adverse Event Reporting System (FAERS): A Focus on Overall Patient Population and Sex-Specific Subgroups. Front. Pharmacol..

[54] Taj S., Zuber M., Rashid M., Chhabra M., Undela K., Rawal S., Villa Zapata L. (2026). Injection-site and Dermatologic Reactions Associated with Glucagon-like Peptide-1 Receptor Agonists: Insights from Meta-analysis of Randomised Controlled Trials and Real-world Evidence. Diabetes Obes. Metab..

[55] Li J., Liang J., Zhang W., He J., Ye X. (2026). Integrated Evidence from VigiBase and Clinical Trials: A Comprehensive Pharmacovigilance Analysis of Seven Glucagon-Like Peptide 1 Receptor Agonists (GLP-1 RAs). Diabetes Ther..

[56] Knop F.K., Aroda V.R., do Vale R.D., Holst-Hansen T., Laursen P.N., Rosenstock J., Rubino D.M., Garvey W.T. (2023). Oral Semaglutide 50 Mg Taken Once per Day in Adults with Overweight or Obesity (OASIS 1): A Randomised, Double-Blind, Placebo-Controlled, Phase 3 Trial. Lancet.

[57] Kadowaki T., Heftdal L.D., Ko H.-J., Overvad M., Shimomura I., Thamattoor U.K., Kim K.-K., Kunishige H., Okude J., Sato N. (2025). Oral Semaglutide in an East Asian Population With Overweight or Obesity, With or Without Type 2 Diabetes. JAMA Intern. Med..

[58] Wharton S., Lingvay I., Bogdanski P., Duque do Vale R., Jacob S., Karlsson T., Shaji C., Rubino D., Garvey W.T. (2025). Oral Semaglutide at a Dose of 25 Mg in Adults with Overweight or Obesity. N. Engl. J. Med..

[59] Eli Lily and Company (2026). Mounjaro Highlights of Prescribing Information.

[60] Laroche M.-L., Géniaux H., Jardou M. (2026). Dysesthesia Associated with GLP-1 Agonist Therapies: Data-Mining Analysis and Literature Review. Eur. J. Clin. Pharmacol..

[61] Ahern S. (2025). Allodynia and Dysesthesia Associated with Semaglutide and Tirzepatide. Cureus.

[62] Stark J., Klass M.J., Owen L. (2025). Allodynia (Skin Tenderness) Associated with Semaglutide: A Case Series. Am. J. Health-Syst. Pharm..

[63] Chakrabarti M.P., Han S., Campbell N.M. (2026). Cutaneous Allodynia Associated With GLP-1RA Tirzepatide for Weight Management: A Case Series. Am. J. Case Rep..

[64] Stokes M., Linnebur S.A., Tietz S., Fixen D.R. (2025). A Case of Semaglutide-Induced Skin Burning. Ann. Intern. Med. Clin. Cases.

[65] Weghuber D., Barrett T., Barrientos-Pérez M., Gies I., Hesse D., Jeppesen O.K., Kelly A.S., Mastrandrea L.D., Sørrig R., Arslanian S. (2022). Once-Weekly Semaglutide in Adolescents with Obesity. N. Engl. J. Med..

[66] Park J.H., Kim J.Y., Choi J.H., Park H.S., Shin H.-Y., Lee J.M., Kim J.-W., Ko H.-J., Chon S., Kim B.K. (2021). Effectiveness of Liraglutide 3 Mg for the Treatment of Obesity in a Real-World Setting without Intensive Lifestyle Intervention. Int. J. Obes..

[67] Bhattarai B., Kadariya B., Al-Shaikhly H. (2025). Allergic Reactions to Anti-Obesity Medications-Analysis of the FAERS Database (Abstract). Ann. Allergy Asthma Immunol..

[68] Ituarte B.E., Taylor M.A., Thomas S.I., Sharma D., High R., Wysong A., Wei E.X. (2024). Cross-Sectional Analysis of Adverse Dermatologic Events Reported to the FDA After Use of GLP-1 Agonists. J. Drugs Dermatol..

[69] Astrup A., Carraro R., Finer N., Harper A., Kunesova M., Lean M.E.J., Niskanen L., Rasmussen M.F., Rissanen A., Rössner S. (2012). Safety, Tolerability and Sustained Weight Loss over 2 Years with the Once-Daily Human GLP-1 Analog, Liraglutide. Int. J. Obes..

[70] Daniel S., Waggett S., Lyles E., Sagut P., Shamaei Zadeh P., Marcelletti A., Stegura C., Wine Lee L. (2025). A Retrospective Comparative Analysis of Cutaneous Adverse Reactions in GLP-1 Agonist Therapies. J. Drugs Dermatol..

[71] Argobi Y., Jadaan N.S., Alshalhoob H.B., Alyousef M.S., Alotaibi G.M., Al Wesaibie H.S., Alduheim M.A. (2026). Skin Manifestations Associated with Glucagon-Like Peptide-1 (GLP-1) Receptor Agonist Use for Weight Loss: A Cross-Sectional Analysis. J. Pak. Assoc. Dermatol..

[72] Chu D., Chen M., Briley J., Salvemini J., Butler D. (2026). Prevalence of Pruritus in Type 2 Diabetic Patients on GLP-1 Agonist Therapy. J. Drugs Dermatol..

[73] Chiang Y.-L., Li T.-H., Fang Y.-W., Liu Y.-F., Wu J., Tsai M.-H. (2026). Dermatologic Outcomes Associated with Glucagon-like Peptide-1 Receptor Agonists in Patients with Type 2 Diabetes: A Large-Scale Target Trial Emulation. Front. Endocrinol..

[74] Saad-Omer S., Kinaan M., Sami N., Mansi I. (2024). 8139 Examining the Association of GLP1-RA or DPP4i with Bullous Skin Diseases in Veterans (Abstract). J. Endocr. Soc..

[75] Hussain H., Ram N., Alarif M. (2026). Semaglutide Induced Bullous Pemphigoid. J. Clin. Transl. Endocrinol. Case Rep..

[76] Burruss C.P., Jones J.M., Burruss J.B. (2021). Semaglutide-Associated Bullous Pemphigoid. JAAD Case Rep..

[77] Collins M.-K. (2021). Bullous Pemphigoid Triggered by Liraglutide. Cutis.

[78] Conway K., Reguram R., Shango K. (2026). Drug-Induced Bullous Pemphigoid Triggered by GLP-1 Receptor Agonist. Endocr. Pract..

[79] Cai S., Zhang Z., Ding W., Zhou S., Qiu X., Hou F., Wu C., Shen Z., Feng X., Jiang L. (2025). The Liraglutide-Induced Pemphigus Vulgaris: A First Case Report Highlighting Autoimmune Risks of GLP-1 Receptor Agonists. Endocr. Metab. Immune Disord. Drug Targets.

[80] Posso-Osorio I., Vargas-Potes C.J., Mejía M., Cañas C.A. (2023). Eosinophil-Related Diseases during Treatment with Glucagon-like Peptide One Receptor (GLP-1 RA): A Case Report and Review of the Literature. Clin. Rheumatol..

[81] Fisher C.M., Wilberg J.M., Rivera S.T., Krauland K.J., Edens C. (2025). Tirzepatide-Induced Lichen Planus Pigmentosus. JAAD Case Rep..

[82] Talasila S., Waseh S., Liu J.Y., Khalifeh I., Metkowski A.R., Hsu S. (2025). Semaglutide-Induced Fixed Drug Eruption. JAAD Case Rep..

[83] Jones T., Myers B., Konia T., Tartar D.M., Rick J. (2025). Granuloma Annulare after Starting Semaglutide. JAAD Case Rep..

[84] Kann R., Weitzner Y., Tan C., Chan C.X., Amin B., Wieder S. (2026). Photodistributed Exfoliative Dermatitis Associated with Semaglutide: Expanding the Spectrum of Glucagon-like Peptide-1 Receptor Agonist–Associated Cutaneous Reactions. JAAD Case Rep..

[85] El Jbeily R., Zhao R., Chaffins M., Matthews N.H. (2026). Symmetrical Drug-Related Intertriginous and Flexural Exanthema in Setting of GLP-1 Agonist: A Case Report. Int. J. Dermatol..

[86] Ouellette S., Frias G., Shah R., Alamgir M., Wassef C. (2023). Dermal Hypersensitivity Reaction to Semaglutide: Two Case Reports. J. Drugs Dermatol..

[87] Saldana-Fraire V., Villareal V., Escalon-Gutierrez E., Pacheco-Estrella D., Paredes A.C. (2025). Desensitization to Tirzepatide after Recurrent Maculopapular Eruptions to GLP-1 Agonists. Ann. Allergy Asthma Immunol..

[88] Moreno-Borque R., Guhl-Millán G., Mera-Carreiro S., Pazos-Guerra M., Cortés-Toro J.A., López-Bran E. (2024). Delayed Type Hypersensitivity Reaction Induced By Liraglutide With Tolerance to Semaglutide. JCEM Case Rep..

[89] Carvallo A., Silva C., Gastaminza G., D’Amelio C.M. (2020). Delayed Hypersensitivity Reaction to Liraglutide: A Case Report. J. Investig. Allergol. Clin. Immunol..

[90] Cogen A.L., Desai K., Elder D., Rosenbach M. (2019). Acute Photodistributed Exanthematous Pustulosis Associated With Liraglutide Treatment. JAMA Dermatol..

[91] Bovijn L., Arianayagam S., Asher R. (2019). An Unusual Case of a Generalised Cutaneous Drug Reaction to Liraglutide. Eur. J. Dermatol..

[92] Paganini C., Belcastro A., Fico A., Talamonti M., Bianchi L., Galluzzo M. (2024). Eczematous Liraglutide Eruption Managed by Dupilumab: A Case Report. JAAD Case Rep..

[93] Santana-Gutiérrez A., Bautista-Gómez A.J., González-Guajardo J.A., Rosales-Solís G.M., Ocampo-Candiani J., Muñoz-Garza F.Z. (2025). Facial Oedema and Necrotic Ulcers: A Cutaneous Reaction to Liraglutide. Clin. Exp. Dermatol..

[94] Goodman W., Pandher K., Ayub M. (2026). Symmetrical Drug-Related Intertriginous and Flexural Exanthema Associated with Tirzepatide. JAAD Case Rep..

[95] Kraft B.D., Matuszak S. (2026). Angioedema After Accidental Semaglutide Dosing Error: A Case Report. J. Clin. Med..

[96] Feinstein J., Hajirawala M., Jalali J., Wild L. (2022). The First Report of Angioedema and Anaphylaxis with Temporal Association to Semaglutide [Abstract]. Ann. Allergy Asthma Immunol..

[97] Barroso B., Gómez-López A., Valverde-Monge M., Betancor D., de las Heras M., Sastre J. (2024). Hypersensitivity Reactions to the GLP-1 Receptor Agonists Liraglutide and Semaglutide: A Case Series. J. Investig. Allergol. Clin. Immunol..

[98] He Z., Tabe A.N., Rana S., King K. (2023). Tirzepatide-Induced Biphasic Anaphylactic Reaction: A Case Report. Cureus.

[99] Nejat C., Lin R., Lee-Wong M. (2025). Clinical Evaluations in Systemic Hypersensitivity to Tirzepatide but Not Semaglutide: Implications for Current Bariatric Treatment [Abstract]. Ann. Allergy Asthma Immunol..

[100] Le T.T.B., Minh L.H.N., Devi P., Islam N., Sachmechi I. (2024). A Case Report of Systemic Allergic Reaction to the Dual Glucose-Dependent Insulinotropic Polypeptide/Glucagon-Like Peptide-1 Receptor Agonist Tirzepatide. Cureus.

[101] Amin T., Mararenko A., Cheng J., Ong R., Holland S., Hu K. (2024). 8348 A Rare Case Of Liraglutide-Associated Angioedema Requiring Emergent Intubation [Abstract]. J. Endocr. Soc..

[102] Harrison K.A., Armstrong S., Vick C., Twede K. (2026). Leukocytoclastic Vasculitis and GLP-1 Agonist: A Case Report. J. Dermatol. Physician Assist..

[103] Pinheiro M.M., de Souza L.G., Nunes G.P., Martin I.F., de Oliveira Y.U., Pinheiro F.M.M., Costa L.N., Caprio M., Della-Morte D., Infante M. (2024). The First Report of Leukocytoclastic Vasculitis Induced by Once-Weekly Subcutaneous Semaglutide. Curr. Med. Res. Opin..

[104] Hoteit M., Worwa S., Koubar S. (2025). IgA Vasculitis Triggered by Semaglutide: SA-PO0910. J. Am. Soc. Nephrol. JASN.

[105] Besemer F., Verschoor A.J., Diamant M., Hoogma R.P.L.M. (2012). Vesiculopustular Dermatosis: An Uncommon Side-Effect of Liraglutide?. J. Diabetes Complicat..

[106] Phelan L.N., Tariq S.F., Singhal R., Wiggins T. (2025). Not Just Skin Deep: A Hidden Complication of Tirzepatide Therapy. Obes. Surg..

[107] Cook S., Selby T. (2025). GLP1 Receptor Agonist Induced Guttate Psoriasis in an Adult Female. SKIN J. Cutan. Med..

[108] Bahar F., Ibis B., Lee Y.-C., Chang K.-Y., Chiang C.H. (2026). Liraglutide May Be Associated with Increased Skin Cancer Risk: A Propensity Score-Matched Analysis. J. Clin. Oncol..

[109] Levy S., Attia A., Elshazli R.M., Abdelmaksoud A., Tatum D., Aiash H., Toraih E.A. (2024). Differential Effects of GLP-1 Receptor Agonists on Cancer Risk in Obesity: A Nationwide Analysis of 1.1 Million Patients. Cancers.

[110] Mitchell P.D., Salter B.M., Oliveria J.P., El-Gammal A., Tworek D., Smith S.G., Sehmi R., Gauvreau G.M., Butler M., O’Byrne P.M. (2017). Glucagon-like Peptide-1 Receptor Expression on Human Eosinophils and Its Regulation of Eosinophil Activation. Clin. Exp. Allergy.

[111] Cote M.F., Oza V.S., Orlow S.J. (2026). Are Pediatric GLP-1 Receptor Agonist Cutaneous Adverse Events Being Overlooked? Emerging Age-Specific Patterns and Their Implications for Dermatologists. Pediatr. Dermatol..

[112] Neel N.F., Ghobara Y., Turkmani M. (2019). Liraglutide-Induced Injection Site Reaction. J. Dermatol. Dermatol. Surg..

[113] Ferrari F., Fierabracci P., Salvetti G., Jaccheri R., Vitti J., Scartabelli G., Meola A., Magno S., Ceccarini G., Santini F. (2020). Weight Loss Effect of Liraglutide in Real-Life: The Experience of a Single Italian Obesity Center. J. Endocrinol. Investig..

[114] Marino-Fernández A.G., García-Gutiérrez I., Alonso Juaristi S., López Gutiérrez J., Tawfiq Piedad M., Guerrero Sotelo Á.L., Albarracín Contreras Á.J., Ortiz Aljaro P., Rodríguez Fernández F. (2025). Hypersensitivity to Liraglutide: A Case Report. Asia Pac. Allergy.

[115] Mizumoto J. (2023). Tirzepatide-Induced Injection Site Reaction. Cureus.

[116] Ayhan I., Topaloğlu Ö., Tekin S., Bayraktaroğlu T. (2023). Injection Site Reaction Induced by Liraglutide Use in a Female Patient with Obesity (Abstract). JCEM Case Rep..

[117] Yaneva M., Handjieva-Darlenska T., Kazandjieva J., Darlenski R. (2023). Delayed Type Hypersensitivity Injection Site Reaction and Tolerance Induction to Liraglutide for the Treatment of Obesity. JAAD Case Rep..

[118] Beshyah S.A. (2025). Balancing Benefit and Risk: Continuing Tirzepatide after Allergic Reactions. Ibnosina J. Med. Biomed. Sci..

[119] Khan D.A., Banerji A., Blumenthal K.G., Phillips E.J., Solensky R., White A.A., Bernstein J.A., Chu D.K., Ellis A.K., Golden D.B.K. (2022). Drug Allergy: A 2022 Practice Parameter Update. J. Allergy Clin. Immunol..

[120] Jaworecka K., Pojawa-Golab M., Otto-Buczkowska E., Reich A. (2019). Injection Site during Liraglutide Therapy. J. Dermatol. Plast. Surg..

