# Supplementary material for: Cutaneous Adverse Events Associated with Semaglutide, Liraglutide and Tirzepatide: A Systematic Review
Source: Med Sci (Basel). 2026 Oct 9;14(6):652. doi: 10.3390/medsci14060652 (PMC13707166; doi:10.3390/medsci14060652)
Supplement: Supplementary file 1 [file medsci-14-00652-s001.zip › medsci-4567526-supplementary-final.pdf]

Study ID	Outcome	D1	D2	D3	D4	D5	Overall
Mastrendrea 2019	Outcome A	!	+	+	+	+	!
Wadden 2023	Outcome A	+	+	+	+	+	+
Jastreboff 2022	Outcome AC	+	+	+	+	+	+
Knop 2023	Outcome B	+	+	+	+	+	+
Wharton 2025 oral	Outcome AB	+	+	+	+	+	+
Lingvay 2025	Outcome AB	+	+	+	+	+	+
Wadden 2021	Outcome A	+	+	+	+	+	+
Wilding 2021	Outcome A	+	+	+	+	+	+
Garvey 2022	Outcome	+	+	+	+	+	+
Wharton 2025 OASIS4	Outcome B	+	+	+	+	+	+
Elkind-Hirsch 2022	Outcome A	+	+	+	+	+	+
Frias 2021	Outcome AC	+	+	+	+	+	+
Rosenstock 2021	Outcome A	+	+	+	+	+	+
Ludvik 2021	Outcome AC	+	+	+	+	+	+
Prato 2021	Outcome A	+	+	+	!	+	!
Danne 2017	Outcome A	+	+	+	+	+	+
Weghuber 2022	Outcome A	+	+	+	+	+	+
Rubino 2022	Outcome A	+	+	+	+	+	+
Kadowaki 2022	Outcome A	!	+	+	+	+	+
Mu 2024	Outcome A	+	+	+	+	+	+
Aronne 2025	Outcome A	+	+	+	+	+	+
Rubino 2021	Outcome A	+	+	+	+	+	+
Kadowaki 2025	Outcome B	+	+	+	+	+	+
Pi-Sunyer 2015	Outcome A	+	+	+	+	+	+
Davies 2015	Outcome A	+	+	+	+	+	+
Wadden 2013	Outcome A	+	+	+	+	+	+
Astrup 2011	Outcome A	+	+	+	+	+	+
Lundgren 2021	Outcome AC	+	+	+	+	+	+
Neeland 2021	Outcome A	+	+	+	+	+	+
Dahl 2022	Outcome A	+	+	+	+	+	+
Inagaki 2022	Outcome A	+	+	+	+	+	+
Kadowaki 2022	Outcome AC	+	+	+	+	+	+

+ Low risk

! Some concerns

\- High risk

D1 Randomisation process

D2 Deviations from the intended interventions

D3 Missing outcome data

D4 Measurement of the outcome

D5 Selection of the reported result

Figure S1. RoB2 for randomized controlled trials.

Study	D1	D2	D3	D4	D5	D6	D7	Overall
Saad-Omer et al., 2024	Very high	Some concerns	High	NA	Some concerns	Some concerns	NA	Very high
Park 2021	High	Low	Some concerns	Some concerns	High	High	Low	High
Argobi 2026	High	Some concerns	Some concerns	Some concerns	NA	High	Some concerns	High
Ferrari 2020	Some concerns	Low	Some concerns	Low	Some concerns	High	Low	High
Disse 2025	Some concerns	Low	Some concerns	Some concerns	Low	Some concerns	Low	Some concerns
Bahar 2026	Low	Some concerns	Some concerns	NA	NA	Low	Low	Some concerns

Domains:
D1: Bias due to confounding.
D2: Bias arising from measurement of exposure.
D3: Bias in selection of participants into the study (or into the analysis).
D4: Bias due to post-exposure interventions.
D5: Bias due to missing data.
D6: Bias arising from measurement of the outcome.
D7: Bias in selection of the reported result.

Judgement
- Very high
- High
- Some concerns
- Low

Figure S2. ROBINS-E for non-randomized observational studies.

Table S1: JBI Critical Appraisal Checklist for Case Reports.

Checklist for case reports (yes- Y, No-N, Unclear-U, Not applicable-N/A)	Were patient's demographic characteristics clearly described?	Was the patient's history clearly described and presented as a timeline?	Was the current clinical condition of the patient on presentation clearly described?	Were diagnostic tests or assessment methods and the results clearly described?	Was the intervention(s) or treatment procedure(s) clearly described?	Was the post-intervention clinical condition clearly described?	Were adverse events (harms) or unanticipated events identified and described?
Burruss	y	y	Y	Y	Y	N/A	N/A
Hussain	Y	Y	y	y	y	N/A	N/A
Collins	Y	Y	Y	Y	Y	N/A	N/A
Conway (A)	y	y	y	y	y	N/A	N/A
Posso-Osorio	y	y	y	y	y	N/A	N/A
Fisher	Y	N	Y	Y	Y	N/A	N/A
Talasila	Y	U	Y	Y	Y	N/A	N/A
Jones	Y	U	Y	Y	Y	N/A	N/A
Kann	Y	Y	Y	Y	Y	N/A	N/A
Jdeily	Y	N	Y	Y	Y	N/A	N/A
Ouellette	y	U	y	y	y	N/A	N/A
Saldana-Fraiare (A)	Y	U	Y	U	Y	N/A	N/A
Moreno-Borque	Y	Y	Y	Y	Y	N/A	N/A
Carvallo	Y	N	Y	Y	Y	N/A	N/A
Cogen	y	y	y	y	y	N/A	N/A
Bovijn	y	y	y	y	y	N/A	N/A
Paganini	y	y	y	y	y	N/A	N/A
Santana-Gutierrez	Y	Y	Y	Y	Y	N/A	N/A
Goodman	Y	Y	Y	N	Y	N/A	N/A
Kraft	y	y	y	y	y	N/A	N/A
Feinstein (A)	Y	N	Y	N	Y	N/A	N/A
Barroso	N	N	Y	Y	Y	N/A	N/A
He	Y	Y	Y	Y	Y	N/A	N/A
Nejat (A)	y	y	y	Y	y	N/A	N/A
Le	y	y	y	U	y	N/A	N/A
Amin	Y	U	Y	Y	Y	N/A	N/A
Harrison	Y	Y	Y	Y	Y	N/A	N/A
Pinheiro	Y	Y	Y	Y	Y	N/A	N/A

Checklist for case reports (yes- Y, No-N, Unclear-U, Not applicable-N/A)	Were patient's demographic characteristics clearly described?	Was the patient's history clearly described and presented as a timeline?	Was the current clinical condition of the patient on presentation clearly described?	Were diagnostic tests or assessment methods and the results clearly described?	Was the intervention(s) or treatment procedure(s) clearly described?	Was the post-intervention clinical condition clearly described?	Were adverse events (harms) or unanticipated events identified and described?
Hoteit	Y	Y	Y	Y	Y	N/A	N/A
Besemer	Y	Y	Y	Y	Y	N/A	N/A
Phelan	Y	U	Y	U	Y	N/A	N/A
Cook	Y	U	Y	U	Y	N/A	N/A
Marino-Fernandez	Y	Y	Y	Y	Y	N/A	N/A
Jaworecka	Y	U	U	N	Y	N/A	N/A
Mizumoto	Y	Y	Y	N	Y	N/A	N/A
Ayhan	Y	Y	Y	N	Y	N/A	N/A
Neel	Y	Y	Y	N	Y	N/A	N/A
Yaneva	Y	U	Y	Y	Y	N/A	N/A
Stoke	Y	Y	Y	Y	Y	N/A	N/A
Ahern	Y	N	Y	N	Y	N/A	N/A
Beshyah	Y	Y	Y	N	Y	N/A	N/A
Chakrabarti	Y	Y	Y	Y	Y	N/A	N/A
Stark	Y	Y	Y	Y	Y	N/A	N/A
